# Interoperable and explainable machine learning models to predict morbidity and mortality in acute neurological injury in the pediatric intensive care unit: secondary analysis of the TOPICC study

**DOI:** 10.3389/fped.2023.1177470

**Published:** 2023-06-28

**Authors:** Neil K. Munjal, Robert S. B. Clark, Dennis W. Simon, Patrick M. Kochanek, Christopher M. Horvat

**Affiliations:** ^1^ Department of Pediatrics, University of Wisconsin—Madison, Madison, WI, United States; ^2^ Department of Critical Care Medicine, UPMC Children’s Hospital of Pittsburgh, Pittsburgh, PA, United States; ^3^ Safar Center for Resuscitation Research, University of Pittsburgh, Pittsburgh, PA, United States

**Keywords:** machine learning, clinical decision support, pediatric intensive care unit, acute neurological injury, predictive modeling

## Abstract

**Background:**

Acute neurological injury is a leading cause of permanent disability and death in the pediatric intensive care unit (PICU). No predictive model has been validated for critically ill children with acute neurological injury.

**Objectives:**

We hypothesized that PICU patients with concern for acute neurological injury are at higher risk for morbidity and mortality, and advanced analytics would derive robust, explainable subgroup models.

**Methods:**

We performed a secondary subgroup analysis of the Trichotomous Outcomes in Pediatric Critical Care (TOPICC) study (2011–2013), predicting mortality and morbidity from admission physiology (lab values and vital signs in 6 h surrounding admission). We analyzed patients with suspected acute neurological injury using standard machine learning algorithms. Feature importance was analyzed using SHapley Additive exPlanations (SHAP). We created a Fast Healthcare Interoperability Resources (FHIR) application to demonstrate potential for interoperability using pragmatic data.

**Results:**

1,860 patients had suspected acute neurological injury at PICU admission, with higher morbidity (8.2 vs. 3.4%) and mortality (6.2 vs. 1.9%) than those without similar concern. The ensemble regressor (containing Random Forest, Gradient Boosting, and Support Vector Machine learners) produced the best model, with Area Under the Receiver Operating Characteristic Curve (AUROC) of 0.91 [95% CI (0.88, 0.94)] and Average Precision (AP) of 0.59 [0.51, 0.69] for mortality, and decreased performance predicting simultaneous mortality and morbidity (0.83 [0.80, 0.86] and 0.59 [0.51, 0.64]); at a set specificity of 0.995, positive predictive value (PPV) was 0.79 for mortality, and 0.88 for mortality and morbidity. By comparison, for mortality, the TOPICC logistic regression had AUROC of 0.90 [0.84, 0.93], but substantially inferior AP of 0.49 [0.35, 0.56] and PPV of 0.60 at specificity 0.995. Feature importance analysis showed that pupillary non-reactivity, Glasgow Coma Scale, and temperature were the most contributory vital signs, and acidosis and coagulopathy the most important laboratory values. The FHIR application provided a simulated demonstration of real-time health record query and model deployment.

**Conclusions:**

PICU patients with suspected acute neurological injury have higher mortality and morbidity. Our machine learning approach independently identified previously-known causes of secondary brain injury. Advanced modeling achieves improved positive predictive value in this important population compared to published models, providing a stepping stone in the path to deploying explainable models as interoperable bedside decision-support tools.

## 1. Introduction

Acute neurological injury is the most common cause of death in critically ill children admitted to the pediatric intensive care unit (PICU) (1, 2). Improvements in the quality of care delivered in PICUs has led to low mortality and shifted attention to morbidity outcomes including survival without new neurological morbidity and long-term neurodevelopment (3, 4). Several studies, primarily in adult and neonatal patients, have demonstrated the burden of neurological morbidity following ICU hospitalization (5–8). Some predictive models have advanced the prediction of mortality in all PICU patients (9); others have focused on a single disease, such as predicting outcomes after severe traumatic brain injury (10). However, there remains a need to predict morbidity and mortality among patients admitted to the PICU with concern for acute neurological injury by all causes.

Machine Learning (ML) approaches have been widely studied in medicine over the past decade (11). They are particularly useful in data-rich environments, which has led to a large amount of investigation in ICU care (12). For patients with acute neurological injury, this approach can leverage newer data sources, such as the use of serum biomarkers of brain injury (13–15). Logistic regression (LR) is the most-used statistical method to model dichotomous outcomes and is a useful technique albeit with some limitations (16). One significant limitation is the difficulty in modeling complex non-linear and non-independent relationships between input variables. Another limitation is the memoryless nature of these models. Approaches to solve the former include Bayesian networks, boosting, support vector machines (SVM), random forest (RF), and neural networks (NN) (16). The latter has been primarily approached with recurrent neural networks and related models. These paths have met with significant success, though their complexity brings about new challenges and sometimes subtle pitfalls that must be carefully addressed in model design (17, 18).

Several validated models have been published to assign mortality risk to patients in the general PICU (19, 20). There are multiple uses for these models: representing severity of illness, monitoring and standardizing quality of care across sites, and acting as surrogates for morbidity and mortality in clinical trials. The incorporation of mortality risk prediction models into a Clinical Decision Support (CDS) tool is often discussed but has not yet been widely adopted. Major reasons for this include the heterogeneity of diseases, the lack of positive predictive value (PPV) in these models due to low mortality and morbidity, the inability to track physiologic evolution over time, and the difficulty to incorporate the effects of intervention. This is particularly true in the setting of acute neurological injury in the PICU, as the standard measures of organ dysfunction (e.g., neurological exam, imaging, electroencephalography) are difficult to distill into straightforward scales (21). The ideal model would allow for real-time automatic monitoring of multiple modalities to better adjust for population illness severity and the subtleties of evolving diseases. However, even a model producing mortality and morbidity estimates on admission has potential value. As a rule-in test, such a tool could bring attention and additional ICU monitoring resources to patients who may be at higher risk than initially apparent, particularly those at intermediate risk. Importantly, an explainable model would help clue the clinician to the associated reasons for concern. While a rule-out test (high sensitivity) may be helpful for reassurance, it is less likely to provide actionable decision support.

We aim to use ML to predict outcomes in PICU patients with concern for acute neurological injury on admission from high-fidelity admission physiologic data collected as part of a multicenter study. We propose that the complex interplay between physiologic variables in PICU patients is better modeled by non-linear ML techniques than previously published traditional LR models, and that these techniques may provide better PPV in predicting patient outcomes. To accomplish this, we applied widely accepted and well-studied ML algorithms to predict outcomes in a cohort of patients with suspected acute neurological injury. We then created a Health Level Seven International (HL7) Fast Healthcare Interoperability Resources (FHIR)-based application to demonstrate an automated vendor-agnostic data pipeline and bedside explainability. We finally explored the use of Shapley Additive ExPlanations (SHAP, RRID:SCR021362) to provide bedside-relevant individual and population-level explanations of model predictions.

## 2. Materials and methods

We performed a secondary analysis of a public-use dataset derived from the Trichotomous Outcomes in Pediatric Critical Care (TOPICC) study (17, 22). TOPICC was a prospective observational cohort study of 10,078 patients under the age of 18 admitted to medical and cardiac ICUs in seven Collaborative Pediatric Critical Care Research Network (CPCCRN) hospitals from 2011 to 2013. The goal of TOPICC was to develop and validate a new predictive instrument for in-hospital mortality and new morbidity based on admission physiologic data (from 2 h before PICU admission, to 4 h after) using the Pediatric Risk of Mortality (PRISM) score. New morbidity was defined as an increase in Functional Status Score (FSS) from admission to hospital discharge by at least 3 points. FSS is a validated age-independent assessment of pediatric function across 6 domains. Each domain is scored from 1 (normal) to 5 (very severe dysfunction), and the total score ranges from from 6 to 30 (22). TOPICC generated a trichotomous model, attempting simultaneous prediction of death, survival with new morbidity, and survival without new morbidity, as well as multiple dichotomous models that combined two of the above outcomes (e.g., survival vs. death or survival without new morbidity vs. death). All categories of included vital signs and laboratory values are listed in Supplementary Table S2.

Patients were selected for a suspicion for acute neurological injury on admission, even if the primary diagnosis was non-neurological; patients who only developed neurological injury during admission could not be ascertained because of the study design. Multiple approaches were attempted to define the subset of patients with acute neurological injury. The PRISM III neurological score, comprised of pupillary reaction and Glasgow Coma Scale (GCS), insufficiently captured the entire population at risk for neurological injury. The admission and discharge diagnosis codes did not capture patients whose primary diagnosis was non-neurological. The TOPICC dataset includes a question asked of the clinician on admission: “Is there reasonable suspicion of possible neurological injury for this patient?” Although subjective, this flag was thought to be most clinically relevant to capture the target population and was used to identify the target subset of patients.

Model selection sought to encompass the breadth of clinically-relevant ML models. The models included were LR, RF, SVM, extreme Gradient Boosting (GB), NN in the form of a Multi-Layer Perceptron (MLP), and an ensemble meta-regressor (23). The LR model mirrored TOPICC with the physiologic data combined into PRISM III scores. Input variables into the LR model included age; admission source; presence of cardiac arrest, cancer, or trauma; primary system of dysfunction; baseline FSS as good/not good; and the PRISM III neurological score and non-neurological score (17). The other ML models were given the raw physiologic variables. Categorical data were included by adding a separate binary yes-no variable for each categorical option, a process called one-hot encoding. The ensemble regressor used the RF, GB, and SVM models as voters in a meta-model. With each of the ML models, bootstrapped train and validation sets were used to prevent overfitting and to obtain statistical distributions for model comparison. Bootstrapping was performed with 50 samples and a sample size fraction of 1.0, drawing elements with replacement and using the out-of-bag elements as the validation cohort. We tested multiple methods for missing data imputation, including median, mode, and k-nearest neighbors imputation; all were single imputation methods. Short model descriptions and final model hyperparameters are included in Supplementary Table S8. Because some algorithms perform poorly on imbalanced data sets, we also attempted oversampling of the target class as well as nearest-neighbor generation of synthetic data, though these techniques did not improve performance in any technique and were not included in the final models, which used median imputation and no class balancing (Supplementary Table S2). Weak calibration testing was performed using least-squares fit of the binned calibration curve and moderate calibration testing was done using calibration plots (24). Strong calibration testing was not conducted due to the low number of patients with the outcome of interest.

Feature importance analysis and model explainability were accomplished with two approaches: standard Gini impurity-based feature importance analysis on the RF and GB model structure, and the use of the SHAP model (25). Both population-level and individual-level explanations were performed. Model parsimony was tested by separate analyses with the top 20, 10, and 3 features as determined by SHAP explanations.

Our statistical analysis of model performance included receiver operating characteristic (ROC) curves and area under the ROC curve (AUROC). Given the imbalanced nature of outcomes in the TOPICC data set, we also included a measure of positive predictive value, so precision-recall analysis was performed using Average Precision (AP), similar to the Area under the Precision-Recall Curve (AUPRC). Precision-recall curves represent the balance between PPV (precision), and sensitivity (recall). Bootstrapped model performance distributions were compared using the Student *t*-test. Determining the optimal cut points for bedside classifier performance requires thoughtful consideration on the parameters to be optimized (i.e., false negative vs. false positive rates), particularly in an imbalanced dataset. To fairly compare models, we reported traditional classifier metrics such as positive and negative predictive value using a cutoff set to guarantee 50% sensitivity, 90% sensitivity, 98% specificity, and 99.5% specificity.

To create an interoperability proof-of-concept, we used an off-the-shelf HL7 Application Programming Interface (HAPI) FHIR server and generated synthetic patient data simulating the first 24 h of admission. All input variables were mapped to Logical Observation Identifiers Names and Codes (LOINC) identifiers. The HAPI FHIR client retrieved patient data to reconstruct the TOPICC variables of highest and/or lowest values in the first 4 h of admission and then fed the preprocessed data into the pre-trained RF model. The web-interface output was the RF and SHAP explainability results.

**Figure 1:**
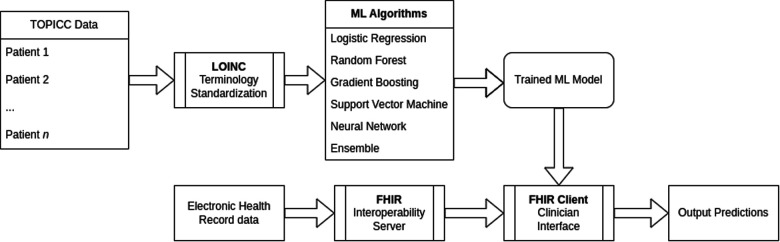
Interoperability and explainability machine learning pipeline.

The full ML pipeline is shown in Figure 1. Software packages included python 3.9.4 (Python Software Foundation, Beaverton, OR, USA; https://www.python.org) with ML packages of scikit-learn 0.24.1, pandas 1.2.3, NumPy 1.20.1, shap 0.39.0, and fhirpy 1.2.1, as well as R 4.0.5 (R Foundation for Statistical Computing, Vienna, Austria; https://www.r-project.org) for model comparison. A HAPI FHIR (5.4.0) backend server was used for the model deployment targeting FHIR R4 4.0.1. All code is available at https://github.com/nkmunjal/PICUNeuroPrediction. TOPICC data, the data used for this study, cannot be shared by the authors but is available by application via the National Institute of Child Health and Development (NICHD) Data and Specimen Hub (DASH, https://dash.nichd.nih.gov/). The sample FHIR client application is available at https://fhirdemo.nkmj.org.

This study was approved by the Institutional Review Board of the University of Pittsburgh on 6/5/2019, approval number STUDY19050230 for the study titled “Secondary Analysis of TOPICC Data using Machine Learning.” As it was a secondary analysis and the primary study was completed, informed consent was waived. Procedures were followed in accordance with the ethical standards of the University of Pittsburgh institutional committee on human experimentation and the Helsinki Declaration of 1975. The Transparent Reporting of a multivariable prediction model for Individual Prognosis or Diagnosis (TRIPOD) checklist is provided as Appendix 1 (26).

## 3. Results

During the original TOPICC study period, 10,078 patients were enrolled. Of those, 1,860 were flagged for suspected acute neurological injury at PICU admission. Their characteristics are summarized in Table 1. Notably, the cohort with suspected acute neurological injury was older but otherwise had similar male predominance, ICU and hospital length of stay compared to the whole cohort. Patients with suspected acute neurological injury had higher mortality (6.2% vs. 1.9%) and new morbidity (8.2% vs. 3.4%).

**Table 1:** Group characteristics of neurological and Non-neurological subset.

Characteristics	Whole TOPICC cohort	No suspected neurological injury	Suspected acute neurological injury
Number	10,078	8,218	1,860
Age (years)	3.7 (0.8–10.8)	3.4 (0.7–10.3)	5.4 (1.3–12.4)
Under 1 year	2,790 (28%)	2,409 (29%)	381 (21%)
Female	4,548 (45%)	3,761 (46%)	787 (42%)
PICU Length of Stay (days)	2 (1–5)	2 (1–5)	2 (1–5)
Hospital Length of Stay (days)	4 (2–10)	4 (2–10)	4 (2–10)
No new morbidity	9,372 (93.0%)	7,779 (94.7%)	1,593 (85.4%)
New morbidity	431 (4.3%)	279 (3.4%)	152 (8.2%)
Mortality	275 (2.7%)	160 (1.9%)	115 (6.2%)

TOPICC, trichotomous outcomes in pediatric critical care study.

**Figure 2:**
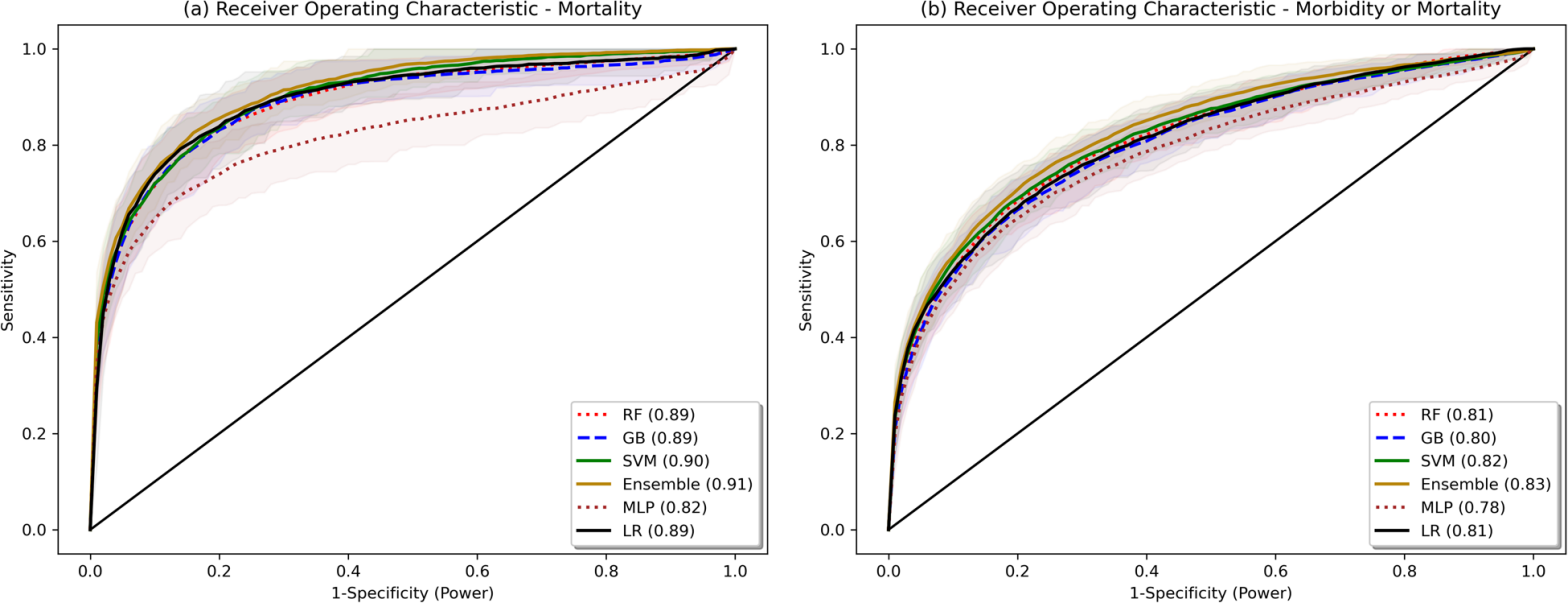
Bootstrapped receiver operating characteristic plots. Shaded area represents bootstrapped 85% Confidence Interval. Bootstrapping was performed with 50 samples, sample size fraction of 1.0, with out-of-bag elements as the validation cohort.

**Figure 3:**
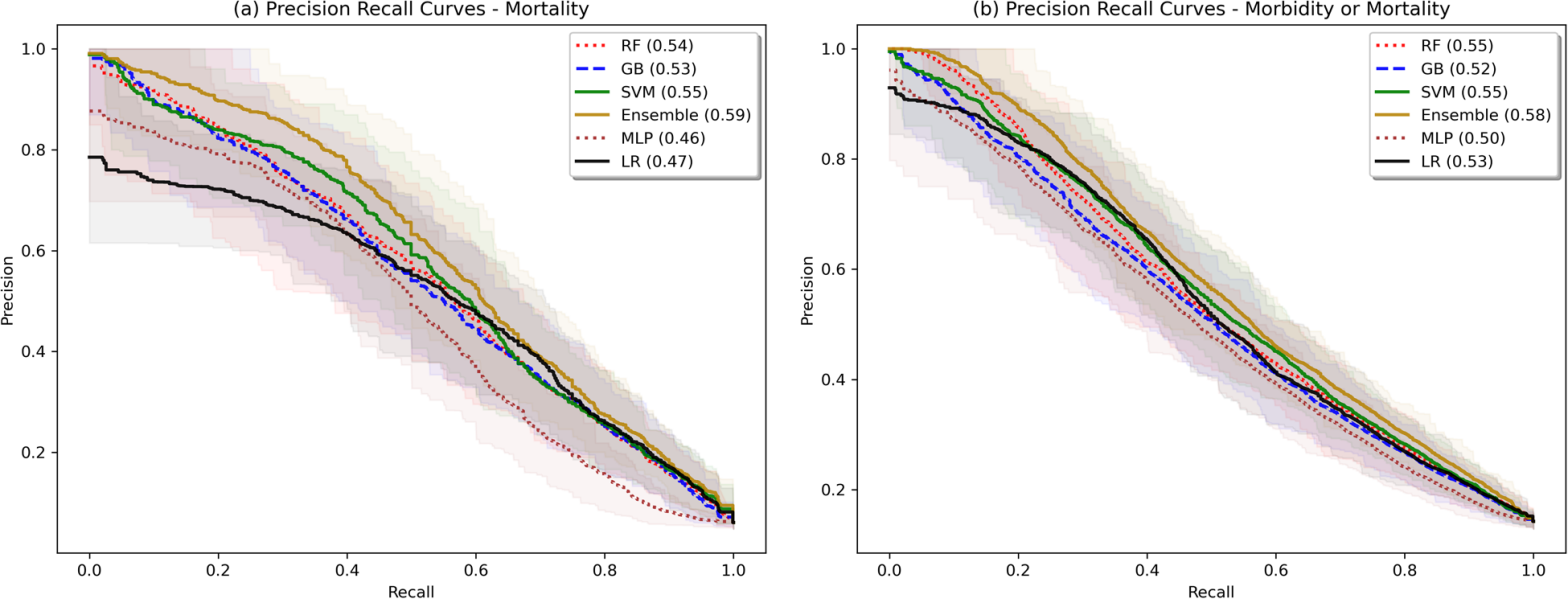
Bootstrapped precision-recall plots. Shaded area represents bootstrapped 85% Confidence Interval. Bootstrapping was performed with 50 samples, sample size fraction of 1.0, with out-of-bag elements as the validation cohort.

The model validation-set results are summarized in Table 2. Standard ROC and precision-recall curves are shown in Figures 2, 3. All models performed better in predicting mortality alone, vs. mortality or new morbidity. The RF and SVM models performed best among base models, with similar AUROC (RF: 0.89 [0.85–0.93 95% bootstrapped confidence interval], SVM 0.90 [0.87–0.93 95% CI]) to the original TOPICC LR model [0.90 (0.84–0.93 95% CI)] and improved AP for mortality (RF: 0.54 [0.41–0.64], SVM: 0.56 [0.44–0.66], LR: 0.49 [0.35–0.56]). For morbidity and mortality, RF, GB, SVM, and LR base models performed similarly. The simple NN model performed poorly. The ensemble learning model improved the PPV of the best performing models [AP: 0.59 (0.51–0.69)], but only after discarding the worst performing model (NN), representing a 10% absolute and 20.4% relative improvement in AP over the original TOPICC LR model performance on this subpopulation.

**Table 2:** Model validation set results.

Predictive model	AUROC (95% bootstrapped confidence interval)	Average precision (95% bootstrapped CI)	Classifier metrics with fixed specificity 0.995 (all 95% bootstrapped CI)		
			Sensitivity	Positive predictive value	Negative predictive value
Mortality versus Survival					
Random Forest	0.89 (0.85, 0.93)	0.54 (0.41, 0.64)	0.29 (0.13, 0.41)	0.73 (0.56, 0.83)	0.96 (0.95, 0.96)
Gradient Boosting	0.89 (0.84, 0.93)	0.53 (0.41, 0.63)	0.28 (0.13, 0.46)	0.75 (0.56, 0.82)	0.96 (0.94, 0.97)
Support Vector Machine	0.90 (0.87, 0.93)	0.55 (0.44, 0.66)	0.32 (0.14, 0.45)	0.76 (0.64, 0.83)	0.96 (0.94, 0.97)
Neural Network (Multilayer Perceptron)	0.82 (0.77, 0.88)	0.46 (0.36, 0.55)	0.24 (0.13, 0.40)	0.71 (0.56, 0.81)	0.95 (0.94, 0.97)
Ensemble	0.91 (0.88, 0.94)	0.59 (0.49, 0.68)*	0.35 (0.25, 0.52)	0.79 (0.69, 0.85)	0.96 (0.95, 0.97)
Logistic Regression	0.89 (0.84, 0.93)	0.47 (0.35, 0.56)	0.15 (0.03, 0.34)	0.60 (0.20, 0.78)	0.95 (0.93, 0.96)
New Morbidity and Mortality versus Survival without New Morbidity					
Random Forest	0.81 (0.77, 0.84)	0.55 (0.47, 0.61)	0.21 (0.14, 0.29)	0.86 (0.82, 0.91)	0.89 (0.86, 0.90)
Gradient Boosting	0.80 (0.75, 0.83)	0.52 (0.44, 0.60)	0.18 (0.10, 0.26)	0.86 (0.79, 0.89)	0.88 (0.86, 0.89)
Support Vector Machine	0.82 (0.78, 0.85)	0.55 (0.46, 0.61)	0.19 (0.11, 0.28)	0.86 (0.77, 0.90)	0.88 (0.86, 0.90)
Neural Network (Multilayer Perceptron)	0.78 (0.75, 0.81)*	0.50 (0.42, 0.57)*	0.15 (0.06, 0.25)	0.82 (0.67, 0.88)	0.87 (0.86, 0.89)
Ensemble	0.83 (0.79, 0.86)	0.58 (0.52, 0.63)*	0.22 (0.14, 0.31)	0.88 (0.82, 0.91)	0.89 (0.86, 0.90)
Logistic Regression	0.81 (0.76, 0.84)	0.53 (0.43, 0.60)	0.18 (0.11, 0.30)	0.85 (0.77, 0.90)	0.88 (0.86, 0.90)

Logistic Regression represents the original study model applied to the neurological concern subpopulation. Sample classifier metrics were derived using a cutpoint defined by setting specificity equal to 0.995, to enable predictive value comparison. AUROC, Area under the Receiver Operating Characteristic curve

* *p* < 0.05 two-tailed compared to Logistic Regression.

For traditional classifier statistics, a cutoff defined to obtain a specificity of 0.995 is shown in Table 2. For mortality, the ensemble had improved sensitivity (ensemble: 0.35 [0.25–0.52], LR: 0.15 [0.03–0.34]), PPV (ensemble: 0.79 [0.69–0.85], LR: 0.60 [0.20–0.78]), and equal NPV. For morbidity and mortality, RF, GB, SVM, ensemble, and LR all performed similarly (ensemble: sensitivity 0.22 [0.14–0.31], PPV 0.88 [0.82–0.91], NPV [0.89 (0.86–0.90)]. Due to class imbalance and fixed specificity, all models had virtually equal negative predictive value (mortality: 0.95, morbidity: 0.88). Additional cutoff comparisons for a set specificity of 0.98, and sensitivity of 0.90 and 0.50, are included in Supplementary Table S1. Notably, there was some separation in PPV between the ensemble and LR at the 0.50 sensitivity cutoff for mortality (ensemble: 0.63 [0.38–0.85], LR 0.55 [0.36–0.72]), but not at the 0.90 cutoff.

Changing the method of oversampling and imputation did not improve the model performance (Supplementary Table S2). The fraction of data points that were missing and required imputation for each feature is listed in Supplementary Table S3. Notably, PaO_2_ was missing from 78% of the subpopulation, Prothrombin Time/Partial Thromboplastin Time (PT/PTT, respectively) from 63%, ionized calcium from 56%, and blood gas values (pH, PCO_2_) from 50%.

**Figure 4:**
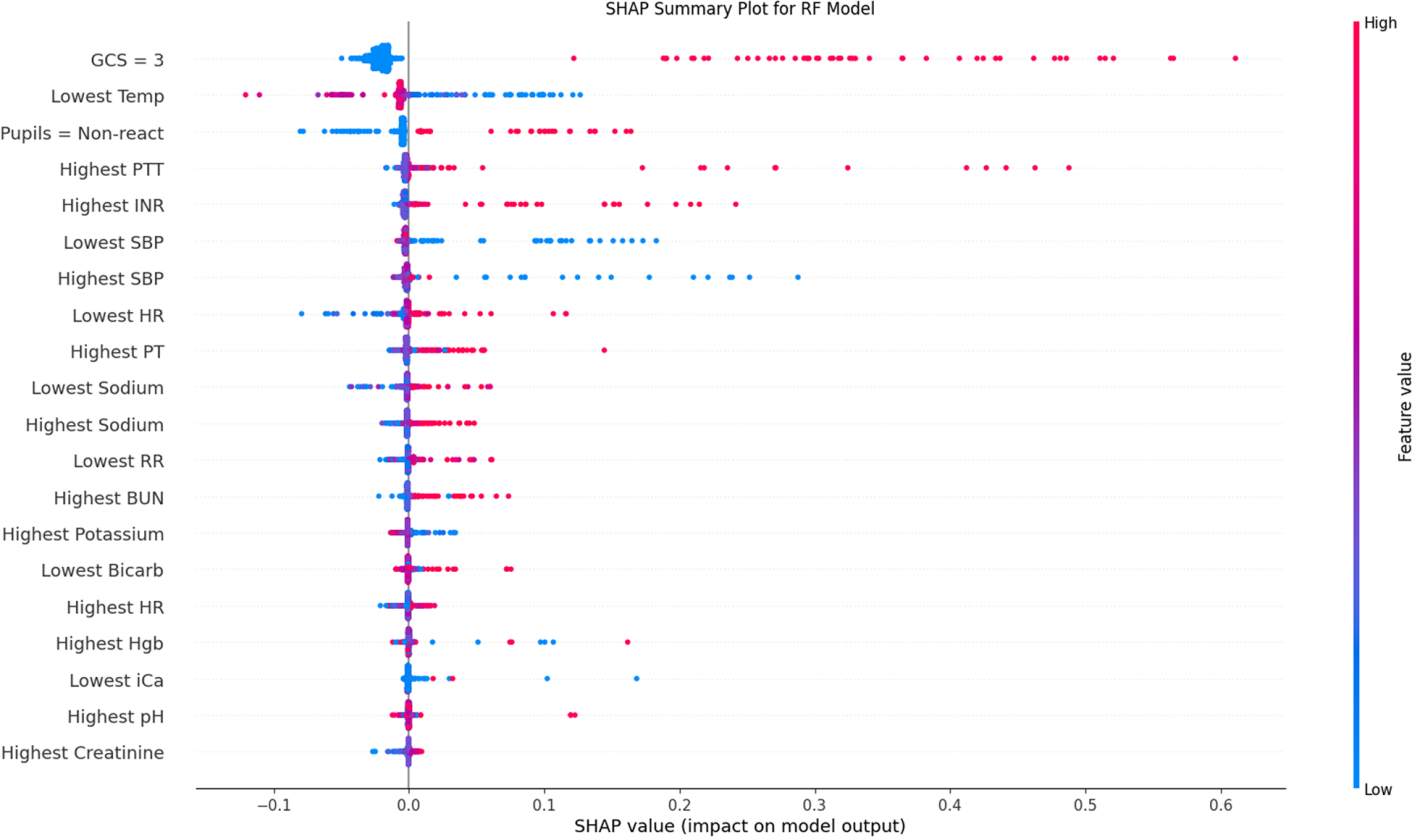
SHAP population-level validation-set explanation plot. Variables are included in descending order by average importance. Red data points represent individual patient values above the mean (continuous variables) or positive (categorical), blue below the mean (continuous) or negative (categorical). Placement on the x-axis is the contribution of that data point towards the final regressor prediction, with more positive values representing higher prediction of mortality. This plot is also frequently called a beeswarm plot. SHAP, SHapley Additive exPlanations; GCS, Glasgow Coma Scale; INR, International Normalized Ratio; PT, Prothrombin Time; PTT, Partial Thromboplastin Time; SBP, Systolic Blood Pressure; HR, Heart Rate; BUN, Blood Urea Nitrogen; Hgb, Hemoglobin; iCa, Ionized Calcium.

SHAP analysis and mean decrease in impurity revealed the most influential features in the RF and GB models. The results for RF Gini feature importance are in Supplementary Table S4 and for the GB feature importance analysis in Supplementary Table S5. The most important features in most bootstrapped models included derangements in temperature, blood pressure, pupillary status, PT/PTT, blood pH, and PCO_2_. Factors that repeatedly were found to be not helpful to the model included sex, hospital site, and intubation status. Figure 4 shows the SHAP summary plot for the RF model, listing the population-wide most important variables explaining the model result in descending order. Categorical variables, such as worst total GCS score and non-reactive pupils are given high importance in the SHAP explanation. Supplementary Figures S1A,B show individual explanatory predictions for patients in whom the model correctly predicted survival without new morbidity (1a) and mortality (1b). Important features for each patient are listed in descending order.

**Figure 5:**
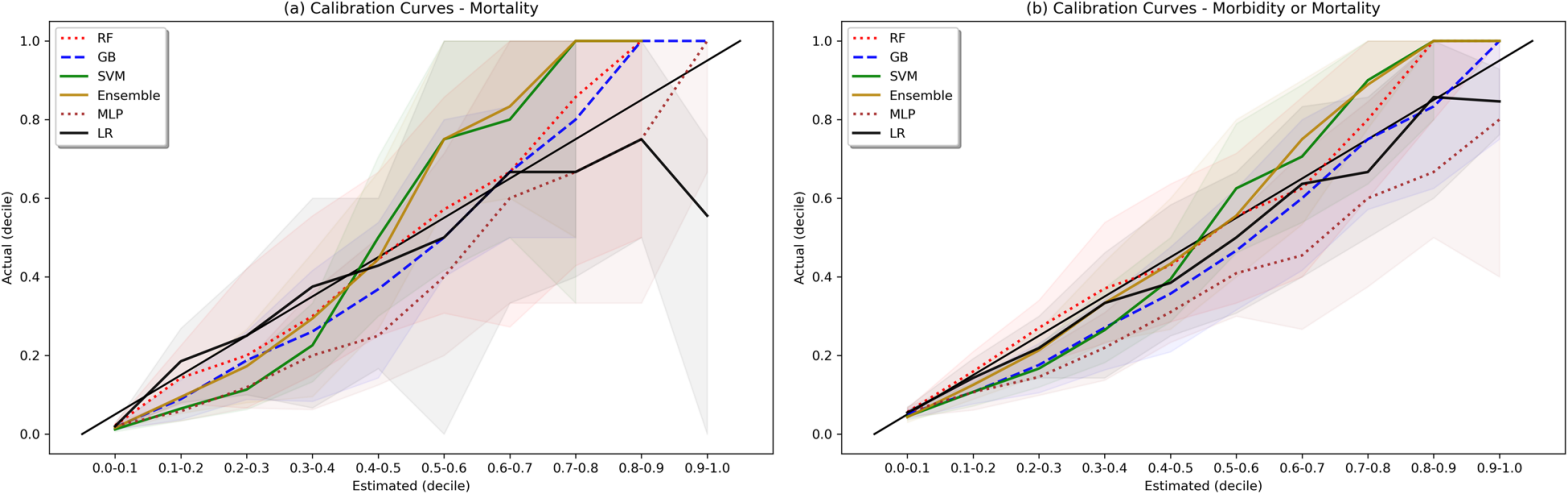
Calibration plots. Shaded area represents bootstrapped 85% Confidence Interval. Ideal calibration occurs when estimated outcome probability is equal to actual outcome fraction for each bin, and is represented by the diagonal line y = x.

Weak calibration statistics are provided in Supplementary Table S6, demonstrating that globally RF and GB had the best least-squared calibration fit, while the SVM, ensemble, and LR models showed global miscalibration in opposite directions. Moderate calibration analysis via calibration curves is shown in Figure 5. For the morbidity and mortality model, all models except NN showed reasonable calibration. For the mortality model, the RF, GB, and Ensemble models show reasonable calibration under 60% and a trend towards underestimating mortality at estimates greater than 60%. The LR model overestimated mortality at estimates greater than 90%, with actual mortality rates around 60%.

The impact of model parsimony on performance is demonstrated in Supplementary Table S7. By taking the top 20 features as determined by the RF model SHAP summary plot we see no change in the predictive accuracy of the RF or GB models. With 10 features, there is a mild average performance loss, and with 3 features, it is marked.

A sample demonstration of a real-time web interface is shown in Supplementary Figure S2. The back-end HAPI FHIR server was pre-loaded with synthetic Electronic Health Record (EHR) patient data simulating PICU admission. The front-end Python FHIR client polls the server in real-time and compiles the admission data to generate a patient row for the pre-trained RF models for mortality alone and mortality and new morbidity. The prediction scores and individual SHAP waterfall plots are then generated and displayed.

## 4. Discussion

We developed and tested multiple ML models to predict both mortality and new morbidity in pediatric patients with suspicion for acute neurological injury on admission to the PICU. Without clinician input on the relative importance of variables, multiple models matched AUROC performance to the original TOPICC-defined PRISM-based LR model, a model with decades of careful generation and refinement, and modestly improved PPV. For this dataset, the RF model and an ensemble meta-model performed best. Though the ROC curves in Figure 2 are similar in appearance (except for the MLP), with an identical mortality/morbidity prevalence between models, the difference in AP/PPV can only come from improvement in sensitivity and/or specificity. With a highly imbalanced dataset, small improvement in sensitivity or specificity can produce greater improvement in PPV, resulting in the modest separation of the precision-recall curves of Figure 3. When evaluating an algorithm for individual-level prediction, rather than population-level prediction, accuracy metrics such as PPV and NPV matter more than sensitivity and specificity, as the clinician does not ask how likely all true positives are test positive, but rather how likely it is that the patient who has a positive test is a true positive. Thus, algorithms should aim to improve PPV or AP, even if the change in AUROC is minimal. In setting fixed sensitivity and specificity cut-offs to provide accurate model comparison, we observed separation between the ML algorithms and LR for the high specificity tests and moderate sensitivity tests (both higher PPV situations) than for the high sensitivity (rule-out) situation. This suggests that LR performs well in many scenarios, but ML may provide advantage in situations where high PPV is needed.

Multiple additional findings merit discussion. First, we used a public use dataset to derive a predictive model for outcome prediction in a neurologically-injured pediatric cohort, a population that does not yet have a population-level predictive model; indeed, high-quality data with this many patients in the PICU is challenging to acquire. Developing such a model is important given the recognized difference in injury burden, whether due to a specific primary Central Nervous System (CNS) problem or secondary to another illness. Second, we attempted to derive this model using advanced algorithms. Given the prevalence of always-accessible computers in modern ICUs, the use of simple decision trees or hand-calculable algorithms must be justified by performance, rather than ease of use. More optimal is to have the most accurate algorithm directly read data from the medical record and easily deliver the result. Third, prediction of morbidity and mortality is more difficult than mortality alone. Given the increasing focus of PICU outcomes on morbidity, this becomes a more critical question. Fourth, this ML study was undertaken with a high-quality prospectively derived dataset, with well-defined input (PRISM physiologic variables) and output (FSS variables). There are multiple advantages to a ML approach with such datasets. First, to a limited degree these algorithms can provide good results requiring minimal data tuning and interpretation. The RF, GB, NN, and ensemble models all used continuous physiologic data, as opposed to the existing TOPICC LR model which required assembly of the data into the previously derived and validated PRISM score. This approach removes some biases; for example, a normal sodium level may be interpreted differently in patients with acute brain injury than those with other diseases, but PRISM (and all other traditional models) has pre-defined cutoffs. LR models can be built to evaluate continuous data as well, and have globally demonstrated non-inferiority to ML models due to increasingly sophisticated augmentative techniques (27). However, as demonstrated in Figure 4, non-linear interactions of variables would not be as easily modelled using LR: for many patients a high hemoglobin was predicted to be protective, whereas for others it was predictive of harm. Without explicitly including the correct interaction between hemoglobin and other variables, LR may not provide a similar result. An important component for any predictive model is the population from which it is derived and for which it is valid. Due to the limitation of the underlying dataset, our use of the flag of suspected neurological injury can both be perceived as a limitation due to its subjective nature, but also as a strength. Given the broad inclusion criteria, our model could be considered valid for any patient that the practicing bedside provider has concern for neurological injury, regardless of their admission diagnosis. Future models may have a more objectively defined population and improved predictive results, but the current model may have sufficient merit to be deployed with this advantage, even implemented as a simple prompt or rule-based trigger embedded in the EHR.

The clinical relevance of our results is notable. Without significant *a priori* clinically-based feature selection, or pre-specified binning of data based on normative values, a sensible set of predictive features was found. In order to be useful as a bedside CDS tool, it is helpful for a model to not only be accurate, but to provide coherent explanations for its prediction to assure the user of its interpretation. Poor GCS score and non-reactive pupils were logically found to be uniformly powerful predictors. Well-recognized secondary CNS insults such as derangements in temperature, blood pressure, coagulation profile, and PCO2 were all demonstrated by both the RF and GB algorithms to be important predictive features. The relationships between variables were not always monotonic in direction or linear in magnitude, producing an advantage afforded to these ML models over LR, which would require explicit inclusion of those interaction terms. Given the diversity of patient diagnoses it is difficult to draw conclusions about direct mechanistic relationships, but our findings may shed light on strategies to identify risk factors secondary brain injury. Though strictly only a measure of association, these tools raise hypotheses about the impact of modifiable risk factors on outcome that could be studied in the future, and potentially even demonstrate the patient population for which it would be most impactful.

Non-linear dynamics are nature's rule, not the exception (28). Complex relationships between variables are more challenging to capture in a linear model such as LR compared to ML models like RF, which outperform LR in scenarios involving expected non-linear interactions (29). For example, the commonly discussed interaction between hypoxemia and hypotension in cerebral oxygen delivery could only be additive in standard LR analysis; these alternative approaches reveal a more nuanced relationship, such as a multiplicative or stepwise effect, and would ideally generate it without supervision.

Model explainability becomes a crucial link to bring predictive models to the bedside (30, 31). As more sophisticated ML models achieve success, we see a natural trade-off in the opacity of the model explanation. Approaches such as the use of mean decrease in Gini impurity for RF models are helpful at the population level, but provide little assistance at the individual level (32). Additionally, Gini impurity also significantly downplays the role that categorical variables (e.g., GCS and pupillary response) have on model output. Recent approaches using simulated local perturbations have improved both aspects of model production (33, 34). In particular, SHAP has become popular due to its ease of use and game-theoretical analytic advantages on both the individual and the population level (35). SHAP models, such as in Figure 4, nicely demonstrate the complexity of these relationships that the ML models have attempted to capture: for example, in some patients a high PaO_2_ was predictive of mortality, while in others a low PaO_2_ was predictive of mortality. High-fidelity explanatory models provide insight into complex physiologic interactions and are a sanity check ensuring the algorithm is learning sensible features in the clinician's mind. Having both population-level and individual-level feature importance is useful, particularly when contemplating future implementation at the bedside. The individual-level predictive graphs seen in Supplementary Figures S1, 2 quickly allow a bedside provider to recognize the prediction model underpinnings, in order of importance.

Even explainable models will only see widespread clinical usage if they are tightly and automatically integrated to a busy ICU workflow (36). Developing interoperable applications was a driving force for HL7 to adopt the FHIR framework (37). FHIR represents a standardized hierarchical data structure whose elements are exposed via a RESTful Application Programming Interface (API). Targeting this API allows for faster vendor-agnostic automated model deployment (38). Interoperability via the FHIR API has become essentially mandatory for all EHR providers via ruling from the Centers for Medicare and Medicaid Services in the United States and is a core feature of National Health Service Digital in the United Kingdom (39). By designing the application with explainability and interoperability at its core, we demonstrate feasibility of clinically-relevant modeling with near-universal automated data collection. The focus of the present models is on admission physiologic data but the FHIR scaffold provides a bridge to automatically collecting data for a future real-time predictive model.

Our study reveals the danger of indiscriminately applying algorithms to a dataset. Each algorithm has a nuanced set of advantages and disadvantages. For example, the RF model easily outperforms the NN in this dataset. There are likely two principal reasons why the NN model underperformed here. First, NNs train best with large volumes of data (often >100 k data points, vs. the 1,860 patients in our cohort) (40). Second, NNs also are susceptible to underperforming with imbalanced datasets, as seen with the 6% mortality rate in our cohort. We attempted oversampling techniques without improvement. For a more straightforward dataset, algorithms that require less training data are more appropriate (41).

Our study has limitations. First, despite the use of bootstrapped train and validation datasets, prospective validation of such models is necessary before clinical use. Second, though the TOPICC data have been well-curated, inconsistencies could still arise when data of this scale are manually entered. Third, as discussed above, such models gain discriminatory power as their numbers increase, so an even larger sample could help with further refinement. Fourth, our use of the subjective “risk for acute neurological injury” flag was limited by the input features in the dataset and certainly makes defining the represented population challenging. Fifth, this model is limited to admission data given the structure of the data source, and therefore causes of permanent injury or death observed during the ICU stay would not be captured by this algorithm. Though the structure of our specific algorithms was optimized for variables from a single time point, similar approaches can be undertaken for continuous monitoring throughout a PICU stay, likely providing greater discriminatory power (9). Additionally, the selected population is likewise limited to patients with suspicion of acute neurological injury on admission. Developing a real-time algorithm validated on patients who develop this concern during their ICU course would also add significant value. Sixth, PPV of 0.5–0.6 may still remain insufficiently high for many clinicians to trust such an algorithm; as a rule-in test, however, a number needed to screen of 2 could also be seen as quite useful if the chosen intervention (e.g., increased ICU monitoring) is relatively low-risk. Finally, it is notable that though the TOPICC dataset provides significant advantages as a prospectively collected trial, the patients were enrolled from 2011 to 2013 and patient characteristics have likely changed in the decade since the last enrolment. In addition, changes in ICU practice and technology have potentially changed outcomes significantly, and possibly heterogeneously with respect to the patient population. These can all potentially result in poorer algorithm performance and miscalibration. Future large-scale data collection would be important to update the algorithm to changes in the patient population and practice.

There are additional limitations from a technical standpoint. First, there were a substantial number of variables with missing data in the original dataset, including 78% who did not have a PaO_2_, and 63% who did not have markers of coagulation. Our multiple attempts at imputation all typically filled in normal values for missing values. This assumption can be problematic, though decision-tree based algorithms still found value in deviations from normal values. Future use of multiple imputation and analytic methods that more accurately understand missingness may be helpful. Second, our observed calibration for some of the models on the mortality task was sub-optimal, both globally (for SVM and ensemble models) and particularly for patients at high estimated risk of mortality. As an example, for patients in the validation set using the ensemble model, if the model gave a predicted output of 0.6, actual mortality was around 0.8. Mis-calibrated models provide a significant challenge if intended for bedside use and can breed mistrust; mis-calibrated accurate models may be trusted less than models with worse performance as measured by AUROC and AUPRC but better calibration (24). Our models for morbidity and mortality were better calibrated, but the ensemble and SVM still demonstrated some global miscalibration. Though we chose not to use a calibration updating corrective algorithm due to concerns about target outcome population size, such an approach could be helpful in future validating studies with larger validation sets.

Finally, it is important to recognize the limitations of population-level predictive models on individual risk prediction. To be useful at the individual level, models need to be accurate, precise, and generalizable to the population of interest. Unfortunately, features that determine population-level risks can be very different than those for the individual, and a model that performs well at the population level may translate poorly to the latter (42). While the TOPICC dataset was generated with the former in mind, we demonstrate improved predictive power in the latter, though potentially not sufficiently precise for current decision support. Further prospective study would be needed to enhance and eventually validate such an individual predictive model.

Our goal was to build an explainable predictive model using a clean and previously-appraised physiologic dataset. Future directions include the application of this approach to real EHR data, the prospective validation of such a model, and the move into real-time monitoring with high-fidelity multi-modal data sources. Each of these steps brings multiple new challenges to carefully address.

## 5. Conclusion

We demonstrate multiple ML approaches that improved positive predictive value over prior PRISM-based LR in predicting both mortality and new morbidity from admission data in patients with risk for acute neurological injury on admission to the PICU. We also identified multiple well-recognized secondary CNS insults as predictive of poor outcomes, supporting biological plausibility. Finally, we demonstrate a proof-of-concept of the need to design models with interoperability and explainability at the core. Future work will focus on the translation of similar algorithms to real-time EHR data and for real-time prediction at the individual level.

## Data Availability

Publicly available datasets were analyzed in this study. This data can be found here: https://dash.nichd.nih.gov/study/226509 - Trichotomous Outcome Prediction in Critical Care.
