# Supplementary material for: Interoperable and explainable machine learning models to predict morbidity and mortality in acute neurological injury in the pediatric intensive care unit: secondary analysis of the TOPICC study
Source: Front Pediatr. 2023 Jun 28;11:1177470. doi: 10.3389/fped.2023.1177470 (PMC10338865; doi:10.3389/fped.2023.1177470)

Supplementary Material

Table of Contents

Supplementary Table 1. Traditional classifier statistics for constant sensitivity and specificity	2
Supplementary Table 2. Oversampling and Imputation results	4
Supplementary Table 3. Data Missingness	5
Supplementary Table 4. Random Forest Gini Feature Importance	6
Supplementary Table 5. Gradient Boosting Feature Importance	7
Supplementary Table 6. Model Calibration	8
Supplementary Table 7. Feature Selection / Model Parsimony	9
Supplementary Table 8. Model Details / Hyperparameters	10
Supplementary Figure 1. SHAP Waterfall plot for 2 patients	11
Supplementary Figure 2. Calibration Curves	12
Supplementary Figure 3. FHIR demo web interface	13

Supplementary Table 1. Traditional classifier statistics for constant a) sensitivity and b) specificity

A) Fixed Sensitivity

**Predictive Model**	Classifier Metrics with Fixed Sensitivity 0.50 (95% bootstrapped CI)			Classifier Metrics with Fixed Sensitivity 0.90 (95% bootstrapped CI)		
	Specificity	Positive Predictive Value	Negative Predictive Value	Specificity	Positive Predictive Value	Negative Predictive Value
**Mortality versus Survival**						
**Random Forest**	0.98 (0.95, 0.99)	0.57 (0.35, 0.73)	0.97 (0.96, 0.98)	0.69 (0.03, 0.80)	0.16 (0.07, 0.25)	0.99 (0.99, 1.00)
**Gradient Boosting**	0.98 (0.95, 0.99)	0.54 (0.37, 0.81)	0.97 (0.96, 0.98)	0.72 (0.56, 0.83)	0.17 (0.12, 0.23)	0.99 (0.99, 0.99)
**Support Vector Machine**	0.98 (0.95, 0.99)	0.61 (0.40, 0.76)	0.97 (0.96, 0.98)	0.71 (0.60, 0.82)	0.17 (0.12, 0.24)	0.99 (0.99, 0.99)
**Neural Network (Multilayer Perceptron)**	0.96 (0.94, 0.99)	0.49 (0.32, 0.70)	0.97 (0.96, 0.98)	0.33 (0.13, 0.62)	0.08 (0.06, 0.14)	0.98 (0.95, 0.99)
**Ensemble**	0.98 (0.95, 0.99)	0.63 (0.38, 0.85)	0.97 (0.96, 0.98)	0.74 (0.61, 0.86)	0.19 (0.13, 0.29)	0.99 (0.99, 0.99)
**Logistic Regression**	0.97 (0.94, 0.99)	0.55 (0.36, 0.72)	0.97 (0.96, 0.98)	0.74 (0.55, 0.85)	0.18 (0.11, 0.31)	0.99 (0.99, 0.99)
**New Morbidity and Mortality versus Survival without New Morbidity**						
**Random Forest**	0.92 (0.88, 0.94)	0.52 (0.40, 0.59)	0.92 (0.91, 0.93)	0.44 (0.29, 0.56)	0.21 (0.17, 0.26)	0.96 (0.94, 0.97)
**Gradient Boosting**	0.92 (0.86, 0.95)	0.51 (0.37, 0.61)	0.92 (0.91, 0.93)	0.42 (0.29, 0.56)	0.21 (0.17, 0.25)	0.96 (0.94, 0.97)
**Support Vector Machine**	0.93 (0.89, 0.96)	0.54 (0.43, 0.67)	0.92 (0.91, 0.93)	0.46 (0.30, 0.59)	0.22 (0.17, 0.26)	0.96 (0.94, 0.97)
**Neural Network (Multilayer Perceptron)**	0.91 (0.86, 0.94)	0.47 (0.38, 0.59)	0.92 (0.90, 0.93)	0.35 (0.21, 0.46)	0.19 (0.15, 0.23)	0.95 (0.92, 0.96)
**Ensemble**	0.93 (0.90, 0.97)	0.56 (0.42, 0.70)	0.92 (0.91, 0.93)	0.50 (0.33, 0.62)	0.23 (0.19, 0.29)	0.97 (0.95, 0.97)
**Logistic Regression**	0.92 (0.88, 0.95)	0.51 (0.36, 0.65)	0.92 (0.91, 0.93)	0.44 (0.32, 0.57)	0.21 (0.17, 0.27)	0.96 (0.95, 0.97)

B) Fixed Specificity

**Predictive Model**	Classifier Metrics with Fixed Specificity 0.98 (95% bootstrapped CI)		
	Sensitivity	Positive Predictive Value	Negative Predictive Value
**Mortality versus Survival**			
**Random Forest**	0.48 (0.34, 0.63)	0.61 (0.48, 0.68)	0.97 (0.96, 0.98)
**Gradient Boosting**	0.47 (0.36, 0.60)	0.61 (0.48, 0.68)	0.97 (0.96, 0.98)
**Support Vector Machine**	0.50 (0.40, 0.61)	0.61 (0.52, 0.70)	0.97 (0.96, 0.98)
**Neural Network (Multilayer Perceptron)**	0.45 (0.36, 0.58)	0.59 (0.50, 0.66)	0.96 (0.95, 0.97)
**Ensemble**	0.51 (0.42, 0.63)	0.62 (0.52, 0.68)	0.97 (0.96, 0.98)
**Logistic Regression**	0.46 (0.33, 0.61)	0.59 (0.50, 0.66)	0.97 (0.95, 0.97)
**New Morbidity and Mortality versus Survival without New Morbidity**			
**Random Forest**	0.31 (0.23, 0.38)	0.71 (0.64, 0.76)	0.90 (0.87, 0.91)
**Gradient Boosting**	0.30 (0.23, 0.41)	0.71 (0.64, 0.76)	0.89 (0.87, 0.91)
**Support Vector Machine**	0.33 (0.25, 0.40)	0.73 (0.65, 0.77)	0.90 (0.88, 0.91)
**Neural Network (Multilayer Perceptron)**	0.28 (0.21, 0.37)	0.70 (0.62, 0.76)	0.89 (0.87, 0.91)
**Ensemble**	0.35 (0.27, 0.41)	0.74 (0.67, 0.78)	0.90 (0.88, 0.92)
**Logistic Regression**	0.33 (0.25, 0.40)	0.73 (0.66, 0.77)	0.90 (0.88, 0.91)

Supplementary Table 2. Oversampling and Imputation results: Random Forest

	**AUROC (95% bootstrapped confidence interval)**	**AUPRC (95% bootstrapped CI)**
	**Mortality versus Survival**	
**Random Forest (RF)**	0.89 (0.85, 0.93)	0.54 (0.41, 0.64)
**RF with 1:1 oversampling**	0.88 (0.84, 0.92)	0.45 (0.34, 0.60)
**RF with *k*-nearest neighbor imputation**	0.89 (0.84, 0.93)	0.53 (0.43, 0.64)
**RF with both**	0.88 (0.84, 0.91)	0.46 (0.31, 0.60)

Supplementary Table 3. Data Missingness

Feature	Fraction of Missing Data
PaO_2	0.78
PT/PTT	0.63
Ionized Calcium	0.56
Blood Gas (pH, PCO_2)	0.50
Complete Blood Count	0.43
Basic Metabolic Panel	0.38
Temperature	0.004
Blood Pressure	0.002
Heart Rate	0
Respiratory Rate	0
Glasgow Coma Scale	0
Pupillary Response	0
Age	0
Sex	0

Supplementary Table 4. Random Forest Gini Feature Importance Analysis

**Highest Importance features**	**Lowest Importance features**
Temperature	Sex
Systolic Blood Pressure	Hospital Site
PTT	White Blood Cell Count
pH	Worst Level of Consciousness
PaO_2	Intubation Status
Age	
PT	
Ionized Calcium	
Heart Rate	

Supplementary Table 5. Gradient Boosting Feature Importance Analysis

**Highest Importance features**	**Lowest Importance features**
Temperature	Sex
Systolic Blood Pressure	Hospital Site
Pupillary non-reactivity	Potassium Level
Heart Rate	Intubation status
Prothrombin Time	Glucose Level
PCO_2	White Blood Cell Count
Ionized Calcium	
Age	
Worst GCS score	
Serum pH	
Anemia	
Sodium level	

Supplementary Table 6. Model Calibration statistics. Weak calibration statistics represent the coefficient and intercept of the least-squares fit for the calibration plot (true probability versus estimated probability). The ideal calibration line has coefficient = 1, intercept = 0. Globally mis-calibrated models will deviate from this line. Values below represent mean and standard deviation of the coefficient and intercept based on the calibration on the validation sets of the bootstrapped samples.

	**Mortality vs Survival**		**New Morbidity or Mortality vs Survival without Morbidity**	
	Coefficient	Intercept	Coefficient	Intercept
**Random Forest**	1.02 +/- 0.22	-0.01 +/- 0.21	1.04 +/- 0.10	-0.00 +/- 0.10
**Gradient Boosting**	1.05 +/- 0.21	-0.06 +/- 0.21	1.01 +/- 0.10	-0.05 +/- 0.10
**Support Vector Machine**	1.25 +/- 0.24	-0.09 +/- 0.24	1.12 +/- 0.11	-0.05 +/- 0.12
**Neural Network**	0.88 +/- 0.15	-0.06 +/- 0.15	0.81 +/- 0.10	-0.02 +/- 0.10
**Ensemble**	1.29 +/- 0.15	-0.08 +/- 0.15	1.18 +/- 0.07	-0.05 +/- 0.07
**Logistic Regression**	0.66 +/- 0.17	0.10 +/- 0.17	0.91 +/- 0.11	0.01 +/- 0.11

Supplementary Table 7. Feature Selection / Model Parsimony. Top features chosen from SHAP beeswarm model for each model type.

	AUROC (95% bootstrapped confidence interval)	AUPRC (95% bootstrapped CI)
**Mortality versus Survival**		
**Random Forest (RF)**	0.89 (0.85, 0.93)	0.54 (0.41, 0.64)
**RF with top 20 features**	0.89 (0.84, 0.93)	0.54 (0.45, 0.65)
**RF with top 10 features**	0.89 (0.85, 0.93)	0.52 (0.40, 0.63)
**RF with top 3 features**	0.77 (0.70, 0.85)	0.42 (0.33, 0.52)
**Gradient Boosting (GB)**	0.88 (0.83, 0.94)	0.53 (0.43, 0.64)
**GB with top 20 features**	0.87 (0.81, 0.93)	0.55 (0.45, 0.62)
**GB with top 10 features**	0.84 (0.79, 0.89)	0.51 (0.41, 0.56)
**GB with top 3 features**	0.66 (0.55, 0.76)	0.29 (0.20, 0.36)

Supplementary Table 8. Model Selection / Hyperparameters. All included models, a brief description of the methodology, and the relevant hyperparameters.

Model	Description	Hyperparameters
**Random Forest**	Decision tree-based voting learner, uses numerous trees each trained using a subset of variables and data points	Number of trees: 150<br>Maximum depth of each tree: 6
**eXtreme Gradient Boosting**	Iteratively improved decision tree based learner, using multiple weak learners that are improved in each iteration and then clustered	Number of learners: 30<br>Learning rate: 0.15<br>Maximum depth: 12
**Support Vector Machine**	Algorithm to determine the optimal "hyperplane" separating the most points in n-dimensional space	Kernel type: Radial Basis Function<br>Kernel degree: 3
**Ensemble meta-learner**	Creates a learner using any arbitrary combination of sub-learners who each have a vote; votes can be uniform or weighted	Learners: RF, XGB, SVM<br>Weights: uniform
**Logistic Regression**	Extension of linear regression, classifies events by creating a formula for the log-odds using a linear combination of the independent variables	
**Multilayer Perceptron (Neural Network)**	Fully connected network of feed-forward artificial neurons, with each neuron having a non-linear activating function.	Network configuration: 2 hidden layers<br>Layer 1 size: 200 neurons<br>Layer 2 size: 50 neurons<br>Alpha: 0.0001

Supplementary Figure 1. SHAP Waterfall (individual-level explanatory) plot for two patients. (a) Explanation for a patient with low predicted mortality, with most important features listed in descending order. (b) Explanation for a patient with high predicted mortality. The value associated with each categorical variable represents the categorical feature (e.g. 0 for no, 1 for yes). The value associated with each continuous variable represents the normalized z-score for the variable, with mean = 0, standard deviation = 1.

SHAP = SHapley Additive exPlanations, GCS = Glasgow Coma Scale, INR = International Normalized Ratio, HR = Heart Rate, PT = Prothrombin Time, iCa = Ionized Calcium, LOC = Loss of Consciousness, SBP = Systolic Blood Pressure

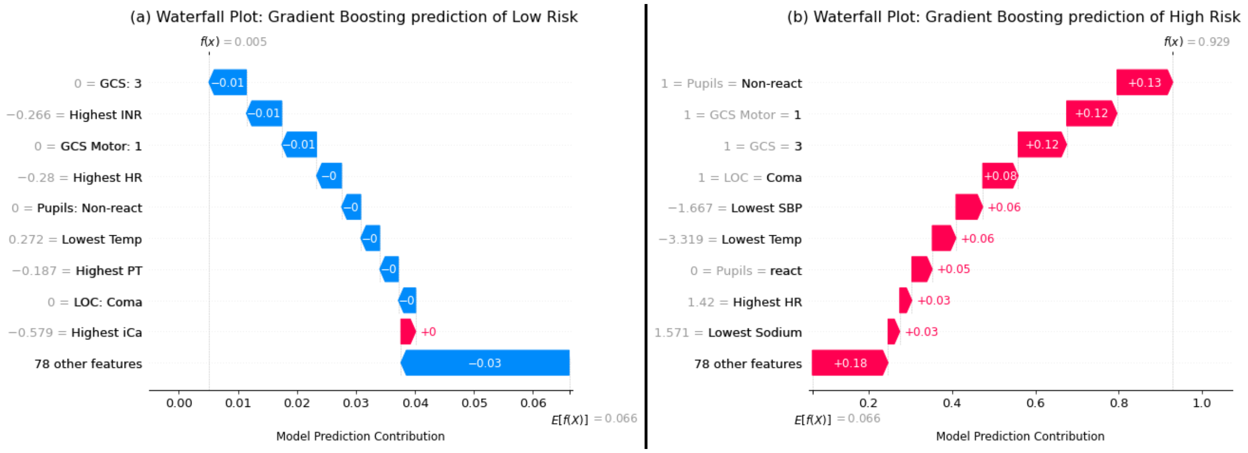

Supplementary Figure 2. FHIR demo web interface. This sample real-time interface allows a clinician to select any active patient and dynamically view the physiologic parameters, as well as the model prediction and SHAP model explanations. It is available at https://fhirdemo.nkmj.org. The value associated with each categorical variable represents the categorical feature (e.g. 0 for no, 1 for yes). The value associated with each continuous variable represents the normalized z-score for the variable, with mean = 0, standard deviation = 1.

TOPICC on FHIR: Home

Patient: Sam Alpha

Admission date: 2021/10/18 14:41:15	
Prediction of mortality:	0.55
Prediction of morbidity or mortality:	0.76
Prediction of return to baseline functional status:	0.24

Category	Variable	Low	High
Demographics			
	Age	7.44	7.44
	Sex	male	
Blood Gas			
	pH	7.11	7.22
	PCO2	36.00	42.00
	PaO2	59.00	60.00
	IonCalcium	0.70	1.18
Metabolic Panel			
	Sodium	155.00	159.00
	Potassium	4.30	4.40
	BUN	13.30	14.20
	Creatinine	1.02	1.04
	Glucose	107.00	143.00
	CO2	26.10	27.90
	TotalCalcium	8.10	9.80
Complete Blood Count			
	WBC	7.70	11.80
	Hemoglobin	9.00	15.20
	Platelets	365.00	367.00
Coagulation Panel			
	PT	25.10	35.00
	PTT	37.40	37.70
	INR	1.10	2.10
Vital Signs			
	Temp	33.00	38.70
	RespRate	12.00	23.00
	HeartRate	68.00	130.00
	SBP	85.00	99.00
	DBP	55.00	90.00
Glasgow Coma Scale			
	GCSMotor	1.00	3.00
	GCSEye	1.00	3.00
	GCSVerbal	1.00	5.00
	GCSTotal	3.00	8.00
	GCSIntub	Patient intubated	
Neuro Exam			
	LOC	Coma (unresponsive)	
	RightPupilResponse	Non-reactive (> 3mm)	
	LeftPupilResponse	Non-reactive (> 3mm)	
	Hypothermia	No	

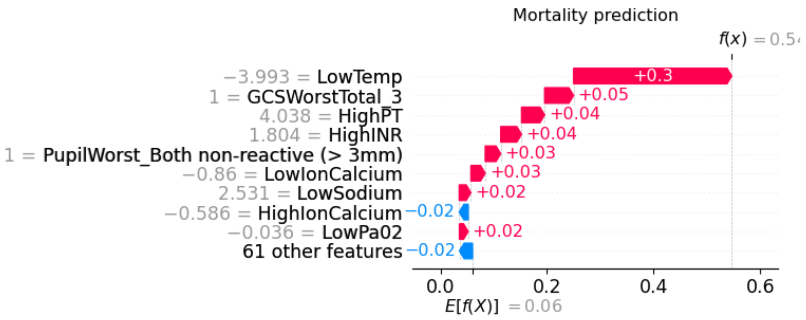

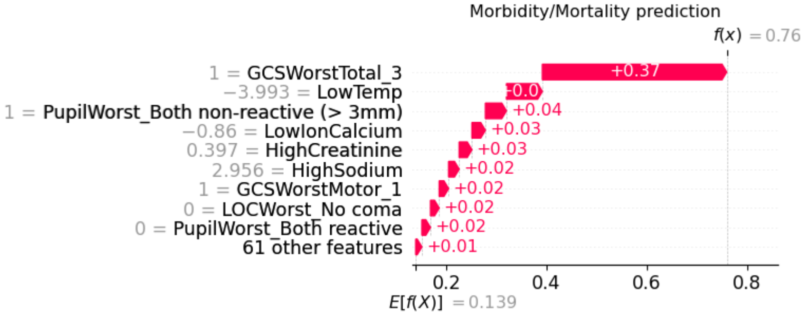

Supplement: Supplementary file 1 [file Datasheet1.pdf]
